# Performance and Mechanism of Functionalized Water Hyacinth Biochar for Adsorption and Removal of Benzotriazole and Lead in Water

**DOI:** 10.3390/ijms24108936

**Published:** 2023-05-18

**Authors:** Pengyang Bian, Qinqin Shao

**Affiliations:** 1 College of Natural Resources and Environment, Northwest A&F University, Xianyang 712100, China; bianpy@nwafu.edu.cn; 2 School of Physics and Electronic Engineering, Zhengzhou Normal University, Zhengzhou 450044, China

**Keywords:** water hyacinth biochar, functional modification, adsorption, benzotriazole, lead

## Abstract

In this paper, water hyacinth is used to prepare biochar (WBC). A biochar–aluminum–zinc-layered double hydroxide composite functional material (WL) is synthesized via a simple co-precipitation method which is used to adsorb and remove benzotriazole (BTA) and lead (Pb^2+^) in an aqueous solution. In particular, this research paper uses various characterization methods to analyze WL and to explore the adsorption performance and adsorption mechanism of WL on BTA and Pb^2+^ in an aqueous solution through batch adsorption experiments combined with model fitting and spectroscopy techniques. The results indicate that the surface of WL contains a thick sheet-like structure with many wrinkles which would provide many adsorption sites for pollutants. At room temperature (25 °C), the maximum adsorption capacities of WL on BTA and Pb^2+^ are 248.44 mg·g^−1^ and 227.13 mg·g^−1^, respectively. In a binary system, during the process of using WL to adsorb BTA and Pb^2+^, compared with that in the absorption on Pb^2+^, WL shows a stronger affinity in the adsorption on BTA, and BTA would thus be preferred in the absorption process. The adsorption process of WL on BTA and Pb^2+^ is spontaneous and is endothermic monolayer chemisorption. In addition, the adsorption of WL on BTA and Pb^2+^ involves many mechanisms, but the main adsorption mechanisms are different. Among them, hydrogen bonding dominates the adsorption on BTA, while functional groups (C-O and C=O) complexation dominates the adsorption on Pb^2+^. When WL adsorbs BTA and Pb^2+^, the coexistence of cations (K^+^, Na^+^, and Ca^2+^) has a strong anti-interference ability, and WL can use a lower concentration of fulvic acid (FA) (<20 mg·L^−1^) to improve its adsorption performance. Last but not least, WL has a stable regenerative performance in a one-component system and a binary system, which indicates that WL has excellent potential for the remediation of BTA and Pb^2+^ in water.

## 1. Introduction

Heavy metals and organic pollutants are increasingly common in water, which has caused severe harm to the ecological environment and human health and has attracted worldwide attention [1]. Lead (Pb^2+^) has always been one of the most polluting metals used in various industries including, inter alia, metal processing, battery manufacturing, and electroplating [2]. Long-term exposure to Pb^2+^ will cause severe damage to the nervous system, reproductive system, and immune system of human body [3]. Even with low concentrations, Pb^2+^ can still cause harm to our health [4]. Benzotriazole (BTA) is usually used as an antifreeze, deicing agent, metal-cutting fluid, antifogging agent, and corrosion inhibitor [5]. This has led to BTA being widely found in our environment. Studies have found that the concentration of BTA detected in some areas is as high as 100 mg·L^−1^ [5]. BTA’s half-life can reach hundreds of days (217–345 d) [6]. In addition, BTA has been considered an emerging pollutant, which has a specific inhibitory effect on the growth and reproduction of aquatic plants and invertebrates and has specific damage to human’s and animal’s skin and respiratory mucosa [7,8]. Even more worrying is that BTA also has the effect of estrogen on the living body [9]. Its metabolized products can interfere with the usual sex differentiation of humans and animals [9]. Due to the wide application of Pb^2+^ and BTA products, the two pollutants are likely to coexist in water bodies, forming more complex pollutants and causing more significant environmental harm. Therefore, removing Pb^2+^ and BTA from water has important practical significance.

Currently, methods for removing heavy metals and organic pollutants from water include catalytic degradation, advanced oxidation, biological treatment, membrane separation, and adsorption [10,11,12]. Adsorption is preferred due to its simple operation, low cost, and generation of few by-products [7]. Currently, there are many studies on the adsorption of Pb^2+^. However, there are few studies on the adsorption of BTA. For example, Zn-Al binary metal oxide has a maximum adsorption capacity of 9.51 mg·g^−1^ on BTA [13]. Pb^2+^ and BTA are likely to coexist, but there are few reports on the competitive adsorption of Pb^2+^ and BTA.

Biochar is a carbon-rich adsorbent obtained by pyrolyzing biomass such as plant residues and animal manure [14]. Biochar is attracting a lot of research attention due to its ability to remove heavy metals and organic pollutants and its stability and potential for catalytic degradation of organic pollutants [15,16]. Its pore structure and functional groups, combined with the wide range of sources for its raw materials, make it a promising material for pollution remediation [17]. Researchers are exploring ways to enhance its adsorption potential, including physical and chemical modifications, and the addition of nanomaterials to its surface. These modifications are expected to improve biochar’s ability to remove pollutants [16].

Layered double hydroxides (LDHs) have received considerable attention over the past few decades [18]. LDH is formed by non-covalent interactions of positively charged sheets containing divalent or trivalent cations with non-skeleton interlayer anions [19]. On the one hand, LDH is rich in hydroxyl groups, which can induce metal ions to produce a deposition effect [20]; on the other hand, LDH is chemically and thermally stable and interpenetrating to different molecules, which contributes to its vital role in removing pollutants in water [21]. However, nanoscale LDH is prone to condensation in water, which reduces the active sites for its adsorption [16]. Studies found that biochar has good dispersion properties and can effectively inhibit the aggregation of particles [22]. Based on this, loading LDH on biochar can not only make up for the defect of LDH being easy to accumulate in the water, but also improve the adsorption performance of biochar and finally achieve a win-win goal.

Water hyacinth is a particularly invasive aquatic plant that has become a severe problem worldwide due to its rapid spread and uncontrolled growth [23]. Research indicates that water hyacinth has been seriously inundated in many eutrophic rivers and lakes in South China, seriously harming aquatic ecosystems and water quality [24]. On the one hand, water hyacinths growing densely on the surface of the water block the river channel and affect navigation and flood discharge [25]; on the other hand, they significantly reduce the concentration of dissolved oxygen and light transmittance in the water, thereby inhibiting the growth of other aquatic plants and animals [26]. Dead remains of water hyacinths would increase the content of nitrogen and phosphorus in the water, which aggravates water eutrophication and leads to the decline of aquatic biodiversity [24]. Therefore, how to properly deal with the flooded water hyacinth is an urgent problem in China. Based on the concept of “treating waste with waste”, converting the biomass of flooded water hyacinth into biochar that adsorbs pollutants in water can help large-scale resource utilization of water hyacinth.

In this paper, water hyacinth is used as raw material to prepare biochar (WBC), and WBC is then functionally modified with Al-Zn-containing layered double hydroxide (LDH), and WL was obtained. The purposes of this research include: (i) characterizing WL and exploring the adsorption effect of WL on Pb^2+^ and BTA in water; (ii) exploring Pb^2+^’s and BTA’s competitive adsorption behavior in a binary system; (iii) exploring adsorption mechanism of WL on Pb^2+^ and BTA in water; and (iv) exploring the regeneration performance of WL when adsorbing Pb^2+^ and BTA. The research results can provide a new reference for the resource utilization of invasive plants and the adsorption removal of heavy metals and organic pollutants in wastewater.

## 2. Results and Discussion 

### 2.1. Characterization of Materials

WBC’s, LDH’s, and WL’s SEM and EDS spectrum images are presented in Figure S1. From the SEM image of WBC (Figure S1a), the surface of WBC is rough, containing more layered stacks and thinner sheets. These sheets are of different sizes, forming more pores. The SEM images of LDH (Figure S1b) show that the surface of LDH is relatively smooth, and its surface contains sheet-like structures. The SEM images of WL (Figure S1c) show that the surface of WL contains a denser sheet structure than that of LDH, and its surface contains a large number of wrinkles. From the EDS spectrum of WL (Figure S1d), the peaks corresponding to the characteristic energies of Zn and Al in WL are firm. However, the Zn/Al ratio is less than 2, which may be caused by a deceased small amount of Zn during the preparation process [27].

The XRD patterns of WBC, LDH, and WL are in Figure S2. The peak corresponding to WBC at position one is caused by K_0.77_Al_1.93_(Al_0.5_Si_3.5_)O_10_(OH)_2_ [28]_,_ and the peak corresponding to position two is caused by SiO_2_ [29]. The corresponding peak of LDH at position three is caused by Al_2_Mg_6_(OH)_16_CO_3_·4H_2_O in the hydrotalcite structure [30]. The XRD pattern of WL contains the peaks caused by K_0.77_Al_1.93_(Al_0.5_Si_3.5_)O_10_(OH) and SiO_2_, and the peaks caused by Al_2_Mg_6_(OH)_16_ CO_3_·4H_2_O in the hydrotalcite structure. This indicates that WL is prepared successfully.

FTIR spectra of WBC, LDH, and WL are presented in Figure S3. The peak at 3347 cm^−1^ results from -OH’s stretching vibration, and the -OH in LDH and WL may be attributed to the bonding between the interlayer water of hydrotalcite and Al-Zn [31]. The peak at 1631cm^−1^ results from C=C’s stretching vibrations in an aromatic structure and C=O’s vibrations in the carbonyl group [32]. Those peaks at 1346 cm^−1^ and 1022 cm^−1^ result from C-N’s and C-O’s stretching vibration [30,33]. While the peak at 785 cm^−1^ is from carbonate ion’s bending vibration [34], the one at 537cm^−1^ is from metal oxide’s variable angle vibration [28].

Pore characteristics of WBC, LDH, and WL are presented in Table S1. Average pore diameters of WBC, LDH, and WL are 10.612 nm, 15.575 nm, and 6.346 nm. This indicates that WBC, LDH, and WL are mesoporous materials [35]. Compared with WBC and LDH, WL provides more adsorption sites for its higher surface area (112.146 m^2^ ·g^−1^) and pore volume (0.184 cm^3^·g^−1^).

### 2.2. Adsorption Effect

**Figure 1:**
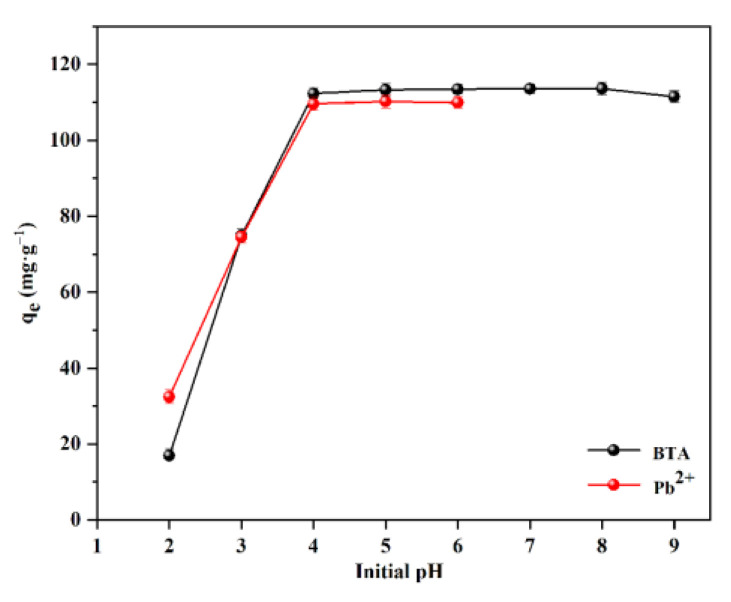
Adsorption Effect of WL on BTA and Pb^2+^ under different pH conditions.

When the pH value of the solution is low (between 2 and 9), Pb is largely in the form of Pb^2+^. When the pH value of the solution is higher than 4, the concentration effect of Pb^2+^ decreases, and Pb mainly exists in the form of [Pb_2_(OH)_3_]^+^ and [Pb_4_(OH)_4_]^4+^. When the pH value of the solution is higher than 6, Pb is mainly in the form of [Pb_4_(OH)_4_]^4+^ and [Pb_3_(OH)_4_]^2+^ [30]. Hence, the pH value of Pb^2+^ solution is adjusted between 2 and 6. The adsorption of WL on BTA and Pb^2+^ under different initial pH conditions is shown in Figure 1. While the pH value is lower, the adsorption capacity effect of WL on BTA and Pb^2+^ is relatively low. For BTA, when the pH value is between 2 and 4, the adsorption amount of BTA by WL increases with the increase in pH value; and when the pH value is between 4 and 8, the adsorption amount of BTA by WL tends to be stable. This is because when the pH value is between 4 and 8, the adsorption capacity of BTA remains at 112.33–113.62 mg·g^−1^. However, when the pH value is higher than 8, the adsorption capacity of WL on BTA decreases. When the pH value is between 2 and 4, the adsorption amount of WL on Pb^2+^ also increases with the increase in the pH value. When the pH value is between 4 and 6, the adsorption amount of Pb^2+^ by WL remains stable at 109.62–110.23 mg·g^−1^.

**Figure 2:**
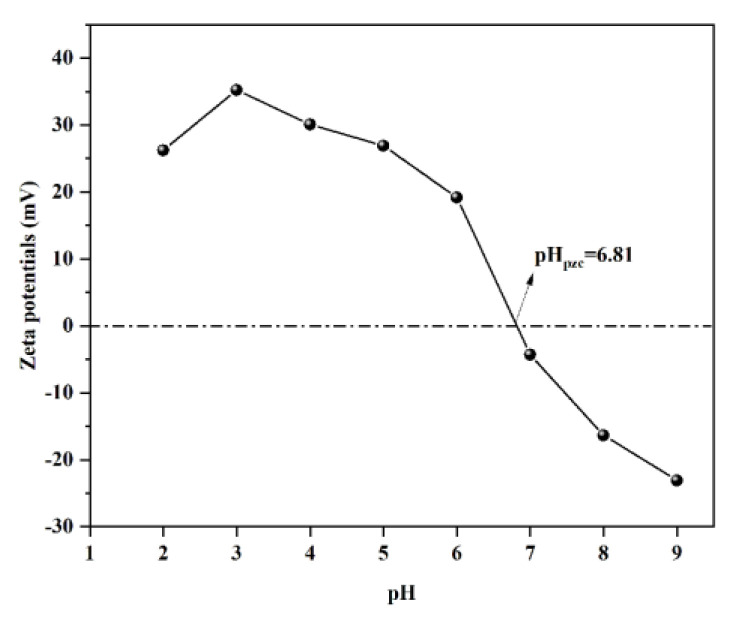
Zeta potential of WL.

The zeta potential of WL is in Figure 2. Zeta potential pH_pzc_ of WL is 6.81, which indicates that when the pH value is higher than 6.81, the surface of WL is negatively charged; and when the pH value is lower than 6.81, the surface of WL is positively charged. Due to electrostatic repulsion, the adsorption of Pb^2+^ by WL should be inhibited. However, when the pH value is between 2 and 4, the adsorption amount of Pb^2+^ by WL increases with the increase in pH value. The phenomenon is similar to the study by Hou et al. [36]. BTA exists as a neutral molecule in the pH range of 1.6–8.3 [13]. Moreover, when the pH is between 2 and 4, the adsorption amount of BTA by WL also increases with the increase in pH value. This indicates that the electrostatic attraction is not dominant in the process of WL adsorption of Pb^2+^ and BTA. It may be that the functional groups in WL plays a leading role in the adsorption process of Pb^2+^ and BTA. As the pH value increases, many functional groups will gradually deprotonate, and the deprotonated functional groups will participate in the Pb^2+^ and BTA adsorption [37,38]. High adsorption capacity of BTA at pH 4–8 may result from hydrogen bonding between hydroxyl groups in WL and imino groups in neutral BTA molecules [13].

### 2.3. Adsorption Kinetics

**Figure 3:**
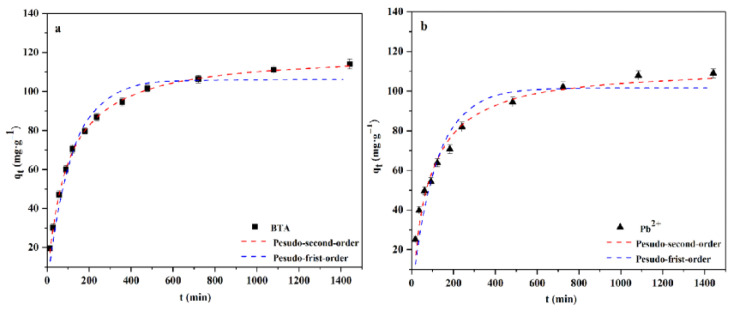
Kinetic model of adsorption of BTA (**a**) and Pb^2+^ (**b**) by WL.

In this study, the pseudo-first-order kinetic and pseudo-second-order kinetic models are used to investigate the adsorption behavior of WL on BTA and Pb^2+^. The former model assumes that adsorbate’s diffusion dominates the adsorption rate to explain the adsorption process, and the entire adsorption process is mainly dominated by physical adsorption [39]. The pseudo-second-order kinetic model assumes that the adsorbent and the adsorbate share or exchange ions/electrons to illustrate the adsorption process. The entire adsorption process is dominated by chemical adsorption [40]. The kinetic model of WL adsorption on BTA and Pb^2+^ is in Figure 3. From Figure 3a,b, we can see that in the first 240 min, the adsorption rates of BTA and Pb^2+^ on WL are faster, which may be related to the mesopores and a large number of active sites in WL [7]. Over time, the adsorption rate gradually slows down to 800 min and the adsorption equilibrium is reached. Table 1 presents the relevant results of the pseudo-first-order kinetic model and the pseudo-second-order kinetic model to the experimental data. The fitting coefficients of pseudo-first-order kinetics to BTA and Pb^2+^ (R^2^) are 0.9711 and 0.8962. The model calculates that when the adsorption reaches equilibrium, the equilibrium adsorption capacities of WL effect on BTA and Pb^2+^ are 107.16 mg·g^−1^ and 102.35 mg·g^−1^, and the pseudo-second-order kinetics to BTA and Pb^2+^ (R^2^) are 0.9989 and 0.9674. When the adsorption reaches equilibrium, the equilibrium adsorption capacities of BTA and Pb^2+^ calculated by the model are 115.34 mg·g^−1^ and 109.02 mg·g^−1^. The calculated values produced by pseudo-second-order kinetics are closer to the experimental values of 114.13 mg·g^−1^ than those of the pseudo-first-order kinetic model one with 106.45 mg·g^−1^. This shows that the adsorption of WL on BTA and Pb^2+^ is more suitable using a pseudo-second-order kinetic model, which also shows the chemical adsorption of WL on BTA and Pb^2+^ [41].

### 2.4. Adsorption Isotherm

**Figure 4:**
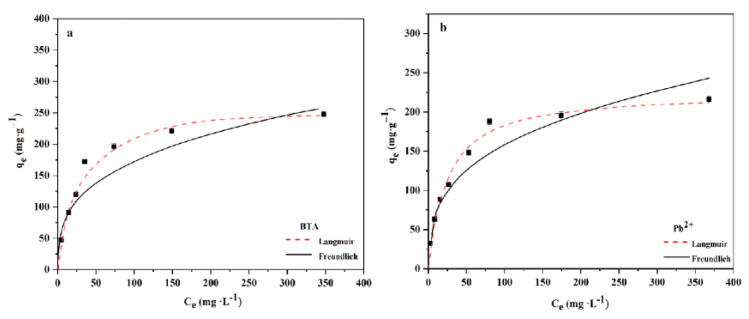
Isotherm model of adsorption of BTA (**a**) and Pb^2+^ by WL (**b**).

This study uses Langmuir and Freundlich isotherm models to fit the adsorption data of WL on BTA and Pb^2+^. The Langmuir model is often used to describe the homogeneous adsorption process. The adsorption sites on a homogeneous surface have an equally effective affinity for adsorbate [42]. The Freundlich model is to depict heterogeneous adsorption of the interaction between the adsorbent and the molecule, and this adsorption process is related to physical adsorption [43]. The adsorption isotherm results of WL on BTA and Pb^2+^ are presented in Figure 4. Increasing BTA and Pb^2+^ concentration means WL’s increased adsorption capacity effect reaches equilibrium. The isothermal model parameters of WL adsorbing BTA and Pb^2+^ are shown in Table 2. The fitting coefficients (R^2^) for the Langmuir model affecting BTA and Pb^2+^ are 0.9884 and 0.9785, while the fitting coefficients (R^2^) for the Freundlich model affecting BTA and Pb^2+^ are 0.8941 and 0.9318. It shows that adsorption of WL on BTA and Pb^2+^ belongs to monolayer adsorption [44]. At 25 °C, maximum adsorption capacity q_m_ of WL on BTA and Pb^2+^ is calculated using the Langmuir model as 248.44 mg·g^−1^ and 227.13 mg·g^−1^. The effect of q _m_ is higher than that of other adsorbents in related reports (Table S2), which indicates that WL has great application potential.

### 2.5. Adsorption Thermodynamics

The effect of temperature on adsorption of WL on BTA and Pb^2+^ is presented in Figure S4a. When temperature increases from 298 K to 318 K, the adsorption effect of WL on BTA and Pb^2+^ increases with increase in temperature, which indicates the increase in temperature is beneficial to the adsorption of WL on BTA and Pb^2+^. Figure S4b,c shows van Hoff curves of WL adsorption on BTA and Pb^2+^. Relevant thermodynamic parameters are presented in Table S3. ΔG° values of WL adsorbing BTA and Pb^2+^ are both negative, which indicates that WL adsorbs BTA and Pb^2+^ and that adsorption reactions are spontaneous [45]. When temperature increases from 298 K to 318 K, ΔG° of WL adsorbing BTA and Pb^2+^ decreases from −30.65 kJ mol^−1^ and −38.15 kJ mol^−1^ to −37.52 kJ mol^−1^ and −41.57 kJ mol^−1^, which further indicates that the higher the temperature, the better the adsorption effect of WL on BTA and Pb^2+^ [46]. ΔH° values of WL absorbing BTA and Pb^2+^ are also positive, which indicates that adsorption is an endothermic reaction [47]. ΔS° values of WL adsorbing BTA and Pb^2+^ are also positive, which indicates absorption of BTA and Pb^2+^ on the solid–liquid interface occurs randomly, and also indicates that the surface structure of WL may interact with BTA and Pb^2+^ via reactive groups [48].

### 2.6. Effects of Coexisting Cations and FA on the Adsorption of WL on BTA and Pb^2+^

**Figure 5:**
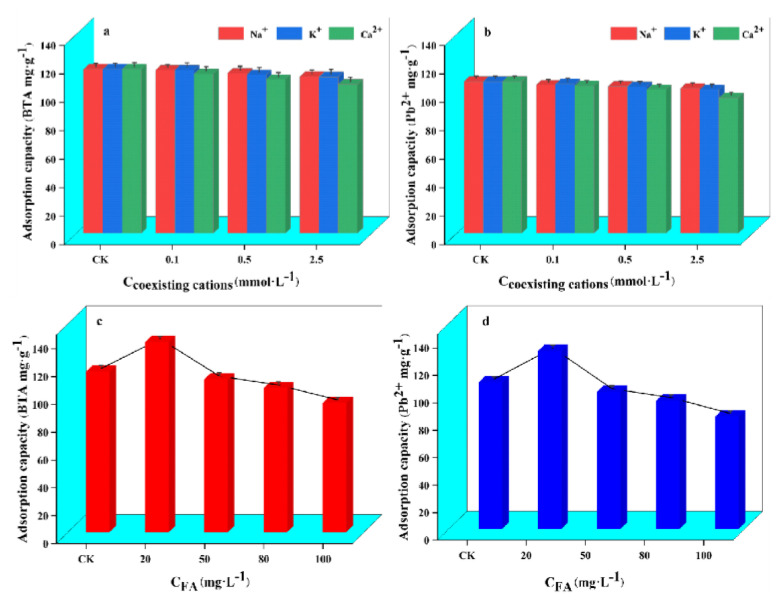
Coexisting cations effect on adsorption of BTA (**a**) and Pb^2+^ (**b**) by WL as well as FA on the adsorption of BTA (**c**) and Pb^2+^ (**d**) by WL.

Since natural water bodies or industrial wastewater usually contain high levels of electrolytes and organic matter, this may affect the adsorption of pollutants by adsorbents [49]. The effect of coexistence of cations and FA on the adsorption of WL on BTA and Pb^2+^ on WL is shown in Figure 5. When no coexisting cation (CK) is added, the adsorption amounts of BTA and Pb^2+^ by WL are 115.32 mg·g^−1^ and 106.72 mg·g^−1^. Compared with CK, WL has a better adsorption effect on BTA and Pb^2+^ in the presence of higher or lower concentrations of coexisting cations. There is no noticeable decline in adsorption, and the reduction ranges are 0.50–9.25% and 2.36–12.06% within the range. This shows that WL has an anti-interference solid ability towards coexisting cations when adsorbing BTA and Pb^2+^, and also shows that ion exchange is not essential in WL adsorbing BTA and Pb^2+^ [50]. From Figure 5c,d, compared with CK, when the concentration of FA is lower (<20 mg·L^−1^), with the increase in FA concentration, the adsorption capacity effect of BTA and Pb^2+^ by WL shows an upward trend. When the concentration of FA is 20 mg·L^−1^, the adsorption capacity of WL on BTA and Pb^2+^ increased by 18.45% and 21.32%. This indicates that a lower concentration of FA will promote the adsorption of WL on BTA and Pb^2+^, which may be due to the presence of a lower concentration of FA that causes a bridging effect between WL and BTA and Pb^2+^, thereby increasing the active site of WL [51]. When the concentration of FA is high (>20 mg·L^−1^), with the increase in FA concentration, the adsorption of BTA and Pb^2+^ by WL shows a decreasing trend, which indicates that higher concentration of FA would inhibit the adsorption of BTA and Pb^2+^ by WL. This may be due to the higher concentration of FA competing with BTA and Pb^2+^ for adsorption sites on the surface of WL or the pores of WL being blocked [28].

### 2.7. Competitive Adsorption of BTA and Pb^2+^

**Figure 6:**
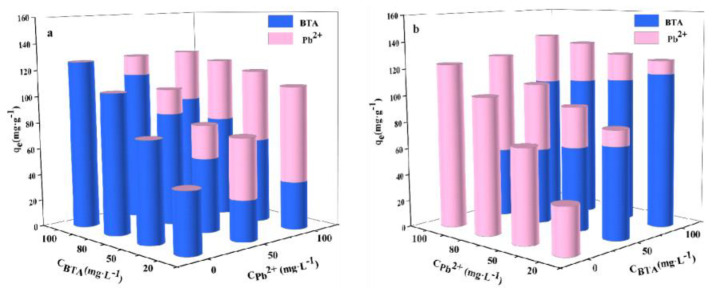
Competitive adsorption of BTA (0–100 mg·L^−1^) (**a**) and Pb^2+^ (0–100 mg·L^−1^) (**b**) by WL.

Competitive adsorption experiments are performed on BTA and Pb^2+^ in a binary system. Competitive adsorption of WL on BTA and Pb^2+^ is presented in Figure 6. When the concentration of BTA is 100mg·L^−1^, and the concentration of Pb^2+^ is 50 mg·L^−1^ and 100 mg·L^−1^, respectively, the adsorption capacity of WL on BTA decreases by about 15.68% and 24.87% accordingly, compared with that in a one-component system. When the concentration of Pb^2+^ is 100 mg·L^−1^ and the concentration of BTA is 50 mg·L^−1^ and 100 mg·L^−1^, respectively, the adsorption capacity of WL on Pb^2+^ decreases by about 32.43% and 61.87% accordingly, compared with that in a one-component system. This result indicates that BTA and Pb^2+^ have a competitive adsorption effect when they are absorbed by WL. When the concentrations of BTA and Pb^2+^ are 50 mg·L^−1^ and 100 mg·L^−1^ simultaneously, the adsorption capacity of WL on BTA is significantly higher. With the increase in Pb^2+^ concentration, the adsorption capacity of WL on BTA remains relatively stable (Figure 6b; and does not appear in Figure 6a). Increasing BTA concentration significantly negatively affects the adsorption capacity of WL on Pb^2+^. This result indicates that when the competitive adsorption effect occurs between BTA and Pb^2+^, WL will have a stronger affinity for adsorbing BTA and it will thus preferentially adsorb BTA. The competitive adsorption of BTA and Pb^2+^ may be caused by the fact that BTA and Pb^2+^ have the same adsorption sites on the adsorbent [50].

### 2.8. Adsorption Mechanism

**Figure 7:**
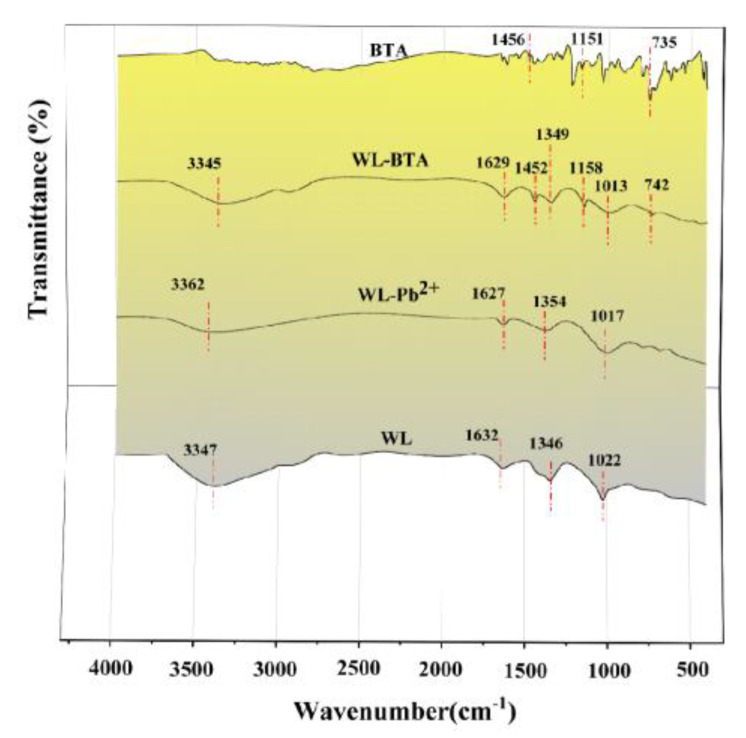
FTIR spectrum of WL adsorbing BTA and Pb^2+^ before and after.

The adsorption process of WL on BTA and Pb^2+^ is complex and involves multiple mechanisms. To better understand how WL absorbs BTA and Pb^2+^, this study utilizes XPS and FTIR analysis on WL before and after adsorption. Figure 7 shows the FTIR spectra of WL before and after adsorbing BTA and Pb^2+^. Figure 7 shows that all the characteristic peaks at 1632 cm^−1^, 1346 cm^−1^, and 1022 cm^−1^ shift after absorption, indicating that functional groups such as C=C/C=O, C-N, and C-O participate in the adsorption process of BTA and Pb^2+^ [50]. The shift in the characteristic peak corresponding to C=C indicates that the Л bond may play an essential role in the adsorption of BTA and Pb^2+^. For example, Pb^2+^ can perform the cation–Л interaction on WL, and BTA can perform the Л–Л interaction on WL [52]. For Pb^2+^, the shift in the characteristic peak corresponding to C=O indicates that Pb^2+^ may have complexation with C=O [53]. The results also indicate that the corresponding characteristic peaks of BTA at 1456 cm^−1^, 1151 cm^−1^, and 735 cm^−1^ shift, which further indicates that BTA is adsorbed by the surface of WL [50]. To sum up, in the process of WL adsorbing BTA and Pb^2+^, the same functional groups are involved, further confirming that there is competitive adsorption between BTA and Pb^2+^.

**Figure 8:**
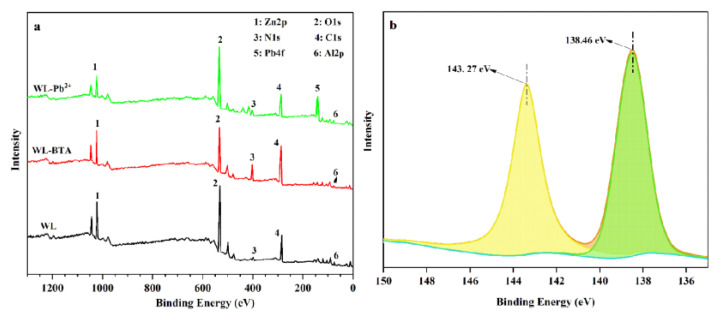
XPS full-spectrum (**a**) of WL diagram before and after (**a**) and the XPS spectrum of Pb4f after WL adsorbing Pb^2+^ (**b**).

The XPS full-spectrum scan of Zn2p, Al2p, C1s, and O1s before and after WL adsorbing BTA and Pb^2+^ is presented in Figure 8. From Figure 8a,b, compared with the XPS spectrum of WL before absorption, the XPS spectrum after the adsorption shows the peak of Pb4f. After the adsorption of BTA, peak intensity of C1s and N1s in the XPS spectrum of WL is enhanced. This result shows that WL has absorbed BTA and Pb^2+^ on its surface. Additionally, Pb4f peaks are further analyzed from Figure 8b. From Figure 8b, we can see that Pb4f shows two peaks at 138.46 eV and 143. 27 eV. The peaks might point to Pb4f_7/2_ and Pb4f_5/2_, which indicate that WL is adsorbing Pb^2+^ and complexes are formed in the process [36]. The binding energy of Pb4f_7/2_ of the peak is located between Pb(OH)_2_ (137.3 eV) and PbCO_3_ (138.7 eV) [54], which indicates that WL is adsorbing Pb^2+^ and two types of Pb-containing compounds are produced.

**Figure 9:**
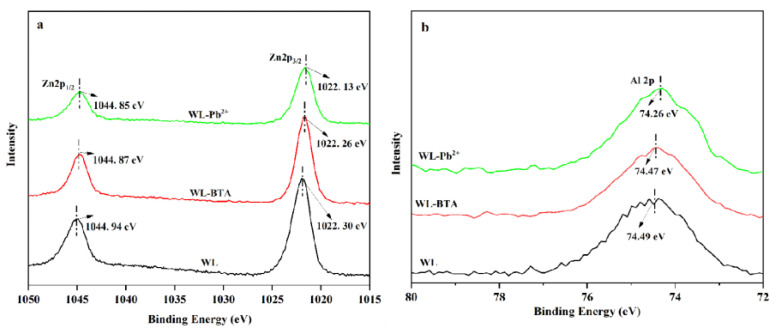

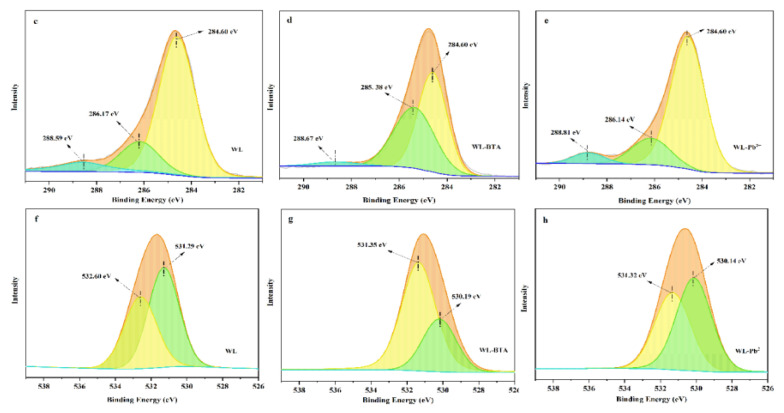
The XPS spectrum of Zn2p, Al2p, C1s, and O1s before and after absorption of BTA and Pb^2+^. (**a**): Zn2p; (**b**): Al2p; (**c**): C1s of WL; (**d**): C1s of WL-BTA; (**e**): C1s of WL-Pb^2+^; (**f**): O1s of WL; (**g**): O1s of WL-BTA; (**h**): O1s of WL-Pb^2+^.

The XPS full-spectrum scan of Zn2p, Al2p, C1s, and O1s before and after WL adsorbing BTA and Pb^2+^ is presented in Figure 9. Compared with the XPS spectrum of WL before adsorption, after WL adsorbing BTA and Pb^2+^, the binding energies of Zn2p and Al2p both decrease, which may be due to the increase in the outer electron cloud density of Al and Zn in WL. Studies have posited that the increase in the outer electron cloud’s density facilitates the pollutants’ adsorption [55]. Figure 9c shows that the C1s of WL before adsorption can be decomposed into three peaks, and the chemical components corresponding to them are C-C at 284.60 eV, C-O at 286.18 eV and C=O at 288.59 eV [56]. From Figure 9d,e, after WL adsorbs BTA and Pb^2+^, the binding energies of the peaks corresponding to C-O and C=O change significantly, but the binding energy of the peaks corresponding to CC does not change significantly. This shows that C-O and C=O are indispensable in getting rid of BTA and Pb^2+^ by WL. Figure 9f shows that the O1s of WL before adsorption can be decomposed into two peaks, and the chemical components corresponding to these two peaks are at C-O at 531.29 eV and C=O at 532.59 eV [57]. From Figure 9g,h, after WL absorbs BTA and Pb^2+^, the binding energies of the peaks corresponding to C-O and C=O shift, which indicates that there is an interaction between C-O and C=O with BTA and Pb^2+^. This finding is consistent with the results of the FTIR analysis.

FTIR and XPS show that the adsorption of BTA and Pb^2+^ involves a variety of interactions, among which the hydrogen bond is essential in the adsorption process of BTA. In contrast, the functional group complexes are indispensable in the adsorption process of Pb^2+^.

### 2.9. Recycling Performance of WL

**Figure 10:**
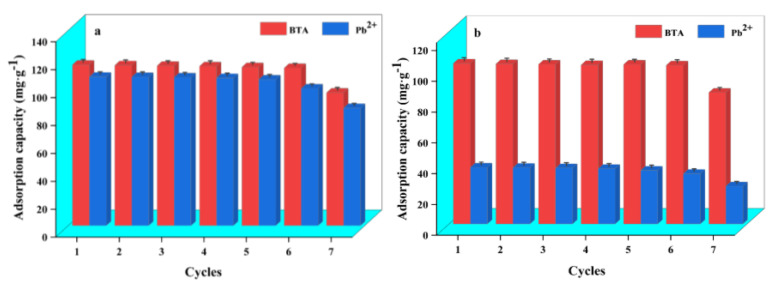
Adsorption–desorption cycle of BTA and Pb^2+^ by WL under a one-component system (**a**) and a binary system (**b**).

The regeneration performance and stability of adsorbents are essential factors in evaluating their potential for practical application. The results of WL recycling experiments are presented in Figure 10. Under a one-component system when WL adsorbs BTA, the adsorption capacity of WL on BTA can still be maintained at 113.02 mg·g^−1^ in the first six-time adsorption–desorption cycles. The adsorption amount of BTA by WL does not decrease significantly until the seven-time adsorption. While under a one-component system, when WL adsorbed Pb^2+^ in the first five-time adsorption–desorption cycles, the adsorption capacity of WL on Pb^2+^ can also be maintained at above 104.76 mg·g^−1.^ After the six-time adsorption, it only decreases by 7.86%. Furthermore, in a binary system, in the first six-time adsorption–desorption cycles, the adsorption capacity of WL on BTA and Pb^2+^ can be maintained at 103.02 mg·g^−1^ and above 32.63 mg·g^−1^. It is not until after the seven-time adsorption that the amount of adsorption of WL on BTA and Pb^2+^ sees a significant decrease by 18.21% and 33.25%. The regeneration experiment results of WL show that regardless of whether in a one-component system or a binary system, WL has a stable regeneration performance, and its effect of the adsorption on BTA and Pb^2+^ can be reused more than six times. Therefore, WL has great application potential in removing BTA in wastewater and Pb^2+^.

## 3. Methods and Materials 

### 3.1. Materials

The water hyacinth in this study is obtained from Changsha, Hunan Province. First, tap water is used to wash away the silt and other impurities on the surface of the water hyacinth after removing unused parts, and the water hyacinth is placed indoors to dry. Then, the air-dried water hyacinth is rinsed with deionized water, and then placed at a 80 °C oven and dried for 24 h. After the water hyacinth is cooled down to room temperature, it is crushed, and then it passes through a 0.149 mm sieve for later use.

All reagents used is of analytical grade. Nitric acid (HNO_3_), sodium chloride (NaCl), potassium chloride (KCl), calcium chloride (CaCl_2_), sodium hydroxide (NaOH), ammonium hydroxide (NH_4_OH), nitric acid hexahydrate Zinc (Zn(NO_3_)_2_·6H_2_O), aluminum nitrate nonahydrate (Al(NO_3_)_3_·9H_2_O, lead nitrate (Pb(NO_3_)_2_), fulvic acid (FA) and BTA is obtained from Shanghai Aladdin Biochemical Technology Co., Ltd. Solutions are prepared from ultrapure water (18.25 ΩΜ) (Nanopure water, Barnstead, NH, USA).

### 3.2. Preparation of Adsorbent

Dried water hyacinth powder is pyrolyzed in a filled-with-nitrogen tube furnace at 600 °C for 5 °C min^−1^ for 2 h. Obtained water hyacinth biochar is marked as WBC. The preparation method of layered double metal hydroxide-modified water hyacinth biochar is based on the works of Olanrewaju et al. [58] and Zhu et al. [30]. The specific steps are: (1) Take 0.005 mol of Al(NO_3_)_3_·9H_2_O and 0.01 mol of Zn(NO_3_)_2_·6H_2_O, and then add 50 mL of ultrapure dissolved water to prepare the cationic precursor solution; (2) Then, take 1.0 g WBC and place it in the cationic precursor solution, thoroughly stir it with a glass rod, and when it forms a uniform suspension, add 0.5M NH_4_OH, and then adjust the pH value of the solution to 8 for co-precipitation; (3) Place the resulting precipitate in a constant temperature shaker at 25 ± 1 °C, shake it at 220 r·min^−1^ for 1 h and then keep it in a water bath at 65 °C for 12 h; (4) Then, rinse the precipitate with deionized water. After washing it several times, place the precipitate in an oven at 60 °C for drying. After drying completely, wait for it to cool to room temperature, put it in a Ziplock bag, and mark the sample as WL. Al-Zn double metal hydroxide (LDH) is prepared by the same method without adding WBC.

### 3.3. Characterization of Samples

Scanning electron microscope (SEM) (JEOL JSM-6700, Tokyo, Japan) is used to characterize the surface morphology of WBC, LDH, and WL. Their elemental compound is then measured using an energy-dispersive X-ray spectrometer (EDS) (QUANTAX400, Bruker, Mannheim, Germany). Crystal composition on the sample surface is then measured using an X-ray diffractometer (XRD) (D8-Advance, Bruker, AXS, Germany). The scanning speed of XRD is 10°·min^−1^ and the scanning range is 5–85°. Fourier transform infrared spectrometer (FTIR) (IRTracer-100, Shimadzu, Tokyo, Japan) is used to characterize sample functional groups. The scanning number of FTIR is 32, the wavenumber range is 400–4000 cm^−1^, and the resolution is 4 cm^−1^. Adsorption–desorption experiments of N_2_ are performed using the Brunauer–Emmett–Teller (BET) (ASAP, 2020 HD88, St. Louis, MI, USA) analysis method, and the BET model determines the surface properties of the samples. The samples are degassed for 2 h before the BET test. X-ray photoelectron spectroscopy (XPS) (Krato AXIS Ultra DLD, Tokyo, Japan) is used to measure chemical and sample surface compounds.

### 3.4. Adsorption Experiment

Detailed description of the adsorption experiment can be found in the Supplementary Materials.

## 4. Conclusions

This study uses water hyacinth as raw material to prepare biochar (WBC). Then, the WBC is functionally modified with Al-Zn-layered double hydroxide (LDH), which then produces a composite functional material (WL). Compared with WBC and LDH, WL has a higher specific surface area (112.146 m^2^·g^−1^) and a denser sheet-like structure. Under a one-component system, the adsorption amounts of WL on BTA and Pb^2+^ are 248.44 mg·g^−1^ and 227.13 mg·g^−1^, respectively. Under a binary system, WL has a stronger affinity for absorbing BTA. The adsorption of WL on BTA and Pb^2+^ is dominated by monomolecular layer chemical adsorption, which is spontaneous and endothermic. The adsorption of WL on BTA and Pb^2+^ involves various interactions, among which hydrogen bonding and functional group complexation are indispensable in the adsorption of WL on BTA and Pb^2+^. When WL adsorbs BTA and Pb^2+^, the coexistence of cations (K^+^, Na^+^, and Ca^2+^) has a solid anti-interference ability, and WL can use a lower concentration of FA (<20 mg·L^−1^) to improve its adsorption performance. Regardless of whether in a one-component or a binary system, adsorption of WL on BTA and Pb^2+^ can be reused more than six times and still has good regeneration performance, which shows that WL has excellent potential for restoring BTA and Pb^2+^ in water.

## Figures and Tables

**Table 1:** Kinetic model fitting parameters of WL to BTA and Pb^2+.^

Pollutants	Pseudo-First-Order			Pseudo-Second-Order		
	K_1_ (min^−1^)	q_e_ (mg·g^−1^)	R^2^	K_2_ (g·(mg·min)^−1^)	q_e_ (mg·g^−1^)	R^2^
BTA	0.0087	107.16	0.9711	0.0003	115.32	0.9989
Pb^2+^	0.0091	102.35	0.8962	0.0001	109.02	0.9674

**Table 2:** Fitting parameters of isotherm model of BTA and Pb^2+^ on WL.

Pollutants	T (°C)	Freundlich			Langmuir		
		K_F_ (L·mg^−1^)	1/n	R^2^	K_L_ (L·mg^−1^)	q_m_ (mg·g^−1^)	R^2^
BTA	25	38.91	0.323	0.8941	0.0352	248.44	0.9884
Pb^2+^	25	35.76	0.324	0.9318	0.3321	227.13	0.9785

## Data Availability

Not applicable.

## Supplementary Materials

The supporting information can be downloaded at: https://www.mdpi.com/article/10.3390/ijms24108936/s1. References [7,28,39,59,60,61,62,63] are cited in supplementary materials.
