# Supplementary material for: Performance and Mechanism of Functionalized Water Hyacinth Biochar for Adsorption and Removal of Benzotriazole and Lead in Water
Source: Int J Mol Sci. 2023 May 18;24(10):8936. doi: 10.3390/ijms24108936 (PMC10219203; doi:10.3390/ijms24108936)
Supplement: Supplementary file 1 [file ijms-24-08936-s001.zip › ijms-2372299-supplementary.pdf]

Detailed de`scription of adsorption experiments

Weigh 1.5986 g of $Pb(NO_3)_2$, dissolve it in 1L of deionized water, and prepare a Pb^{2+} stock solution, and then the stock solution was diluted to 100mg·L^{-1}. Similarly, prepare BTA solution of a concentration of 100 mg·L^{-1}. In this study, the preliminary experiments confirmed that the optimum dosage of WL adsorbing BTA and Pb^{2+} is 0.035g. Hence, the dosage of WL in the batch adsorption experiments is 0.035g.

(1) pH experiment: To explore the effect of pH on the adsorption performance of WL by adjusting the initial pH value of the solution: take 50mL of Pb^{2+} and BTA solution with a concentration of 100 mg·L^{-1} and place it in a set of 100mL centrifuge tubes; and use a solution of 0.1mol·L^{-1} HNO_3 and NaOH to adjust the pH of the Pb^{2+} solution between 2-6, and the pH of the BTA solution is adjusted at 2-9; later, 0.035g WL sample is added to Pb^{2+} and BTA solution respectively, then they are shaken in a constant temperature shaker at 25±1℃at 220 r·min^{-1} for 24h. They are then centrifuge at 4000 r·min^{-1} for 10 mins, and the supernatant is taken and then filtered it through a 0.45μm GE cellulose nylon membrane. The content of Pb^{2+} in the filtrate is determined by inductively coupled plasma mass spectrometer (ICP-MS, NexION 2000, Perkin-Elmer SCIEX, USA). The content of BTA in the filtrate is determined by a UV-Vis spectrophotometer (UV-2550, AOE, China). Three replicates are set up for each treatment. According to the contaminants (Pb^{2+} and BTA) concentration difference to calculate WL adsorption of Pb^{2+} and BTA content. The formula is as follows:

$$q_e = \frac{(C_0 - C_e) \times V}{m} \quad (a)$$

In the formula, q_e (mg·g^{-1}) represents the adsorption amount of WL when the adsorption reaches equilibrium; C_0 (mg·L^{-1}) and C_e (mg·L^{-1}) represent pollutants (Pb^{2+} and BTA) initial concentration and equilibrium concentration; V(L) is the solution volume; m(g) is the quality of WL.

(2) Adsorption kinetics: Take 50mL of Pb^{2+} and BTA solution respectively with a concentration of 100 mg·L^{-1} and place them in a set of 100mL centrifuge tubes, and then 0.035g WL is added, and then the centrifuge tubes are placed in a constant temperature shaker at 25±1℃ and oscillated at 220 r·min^{-1}, respectively. Samples are taken at different time points (5-1440min). Then the remaining pollutant concentration in the solution (Pb^{2+} and BTA) are taken. Filtration of solutions, centrifugation and determination of pollutant concentrations and the method of the pH experiment in (1) are the same. Three replicates were set up for each treatment. Fitting analysis of experimental data was performed using the pseudo-first-order kinetic model (b) and pseudo-second-order kinetic model (c). The formula is as follows:

$$\ln(q_e - q_t) = \ln q_e - k_1 t \quad (b)$$

$$\frac{t}{q_t} = \frac{1}{k_2 q_e^2} + \frac{t}{q_e} \quad (c)$$

In the formula, q_t and q_e (mg·g $^{-1}$) represents the adsorption capacity at time t and adsorption equilibrium, and K_1 and K_2 represent the rate constants of the pseudo-first-order kinetic and pseudo-second-order kinetic models, respectively.

(3) Adsorption isotherm: take 50mL of different concentrations of Pb^{2+} and BTA solutions (10 -500 mg·L^{-1}) and place them in 100mL centrifuge tubes. Then 0.035g WL is added, and the centrifuge tubes are placed in a constant temperature shaker at 25±1℃, and Oscillated at 220 r·min^{-1} for 24h. Take samples. Then measure the remaining pollutant concentrations in the

solution (Pb^{2+} and BTA). The filtration, centrifugation and determination of pollutant concentration of the solution are the same as the method of pH experiment in (1). Three replicates were set up for each treatment. The analytical data were fitted using Freundlich (d) and Langmuir (e) isotherm models. The formula is as follows:

$$q_e = K_F C_e^{1/n} \quad (d)$$

$$q_e = \frac{C_e q_m K_L}{1 + C_e K_L} \quad (e)$$

In the formula, where K_F represents the adsorption constant of the Freundlich isotherm model, 1/n represents the adsorption intensity parameter of the Freundlich isotherm model, dimensionless; C_e represents the pollutant (Pb^{2+} and BTA) concentration at adsorption equilibrium ($mg{\cdot}L^{-1}$), q_m is the maximum adsorption capacity of WL ($mg{\cdot}g^{-1}$), andK_L represents the adsorption constant of the Langmuir temperature model.

(4) Adsorption thermodynamics: at different temperatures (25℃, 35℃and 45℃), explore the adsorption capacity effect of Pb^{2+} and BTA on WL, and investigate the thermodynamics of adsorption of Pb^{2+} and BTA on WL. Use the Gibbs-Helm Holtz Equation to study the thermodynamics of adsorption of Pb^{2+} and BTA on WL. The formula is as follows:

$$\Delta G^{\circ} = -RT\ln K_d \quad (f)$$

$$K_d = q_e\, m / C_e\, V \quad (g)$$

$$\Delta G^{\circ} = \Delta H^{\circ} - T\Delta S^{\circ} \quad (h)$$

In the formula, ΔH° ($J{\cdot}mol^{-1}$) represents enthalpy change; ΔS° ($J{\cdot}mol^{-1}{\cdot}k^{-1}$) represents entropy change; ΔG^{o} ($kJ\ mol^{-1}$) represents Gibbs free energy; K_d is the adsorption partition coefficient R (8.314 $J{\cdot}mol^{-1}{\cdot}k^{-1}$) represents the gas constant; T(k) stands for absolute temperature.

(5) Competitive adsorption of BTA and Pb $^{2+}$: set the concentration of BTA to 20 $mg{\cdot}L^{-1}$, 50 $mg{\cdot}L^{-1}$, 80 $mg{\cdot}L^{-1}$ and 100 $mg{\cdot}L^{-1}$, respectively. Explore different concentrations of Pb^{2+} (0 $mg{\cdot}L^{-1}$, 50 $mg{\cdot}L^{-1}$, 100 $mg{\cdot}L^{-1}$) on the adsorption of BTA by WL. Similarly, the Pb^{2+} concentrations are set to 20 $mg{\cdot}L^{-1}$, 50 $mg{\cdot}L^{-1}$, 80 $mg{\cdot}L^{-1}$ and 100 $mg{\cdot}L^{-1}$, and different concentrations of adsorption of Pb^{2+} on WL are explored in the presence of BTA at different concentrations (0 $mg{\cdot}L^{-1}$, 50 $mg{\cdot}L^{-1}$, 100 $mg{\cdot}L^{-1}$).

(6) Effects of coexisting ions and HA on the adsorption of BTA and Pb^{2+} on WL: the common cations in water (Na^{+}, K^{+} and Ca^{2+}) as a representative to explore the effect of adsorption of coexisting ion pairs of BTA (100 $mg{\cdot}L^{-1}$) and Pb^{2+} (100 $mg{\cdot}L^{-1}$). In this study, the concentrations of these cations were set between 0-2.5$mmol{\cdot}L^{-1}$. HA is the main component of natural organic matter (NOM) in natural water. The concentration of HA is set at 0-100$mg{\cdot}L^{-1}$ to explore the effect of absorption of organic matter in water on BTA (100 $mg{\cdot}L^{-1}$) and Pb^{2+} (100 $mg{\cdot}L^{-1}$).

(7) Recycling experiment of WL: the used WL will be collected. 95% ethanol and 0.1 $mol{\cdot}L^{-1}$ HCl as adsorbents will be used respectively to desorb BTA and Pb^{2+} from WL. Then the regenerated WL after washed three times with deionized water and dried. Then the reusability of WL will be explored.

In experiments (5), (6) and (7), each treatment also set three repetitions. Shaking, filtering, centrifuging and determining pollutants concentrations (Pb^{2+} and BTA) are completed with using the same the method of the pH experiment in (1).

Figure captions

Figure S1 SEM images of WBC (a), LDH (b), WL (c) and EDS spectrum of WL (d)

Figure S2 XRD pattern of WBC, LDH and WL

Figure S3 FTIR spectra of WBC, LDH and WL

Figure S4 Effect of temperature versus WL adsorption BTA and Pb^{2+} (a); WL adsorption of BTA (b) and Pb^{2+} (c) Van't Hoff curve

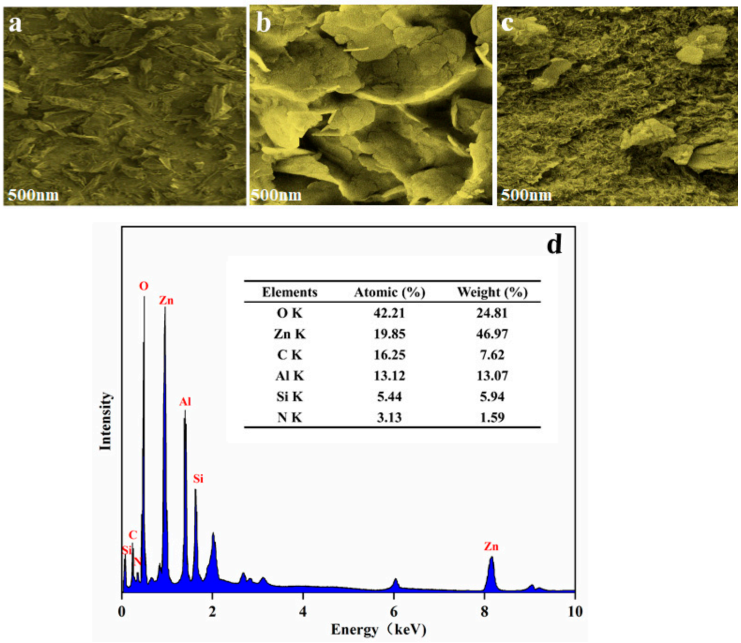

Figure S1 SEM images of WBC (a), LDH (b), WL (c) and EDS spectrum of WL (d)

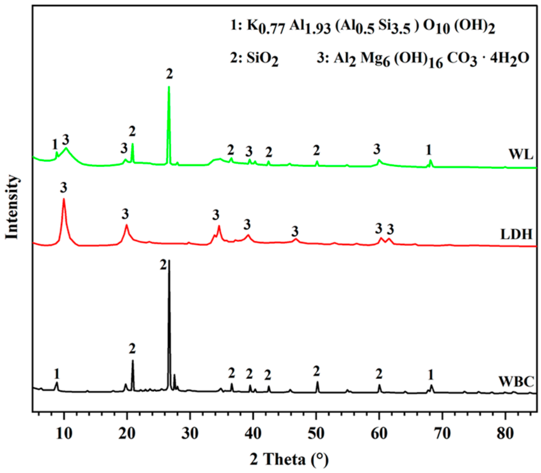

Figure S2 XRD pattern of WBC, LDH and WL

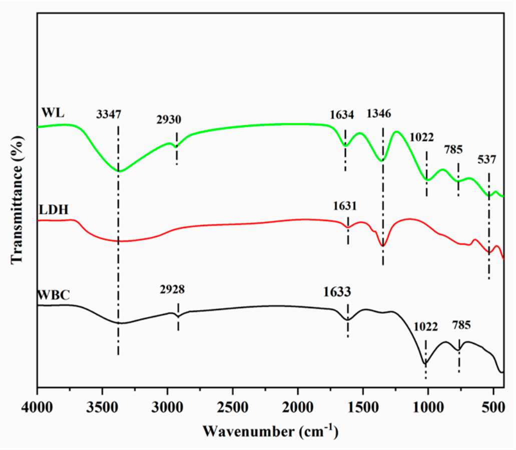

Figure S3 FTIR spectra of WBC, LDH and WL

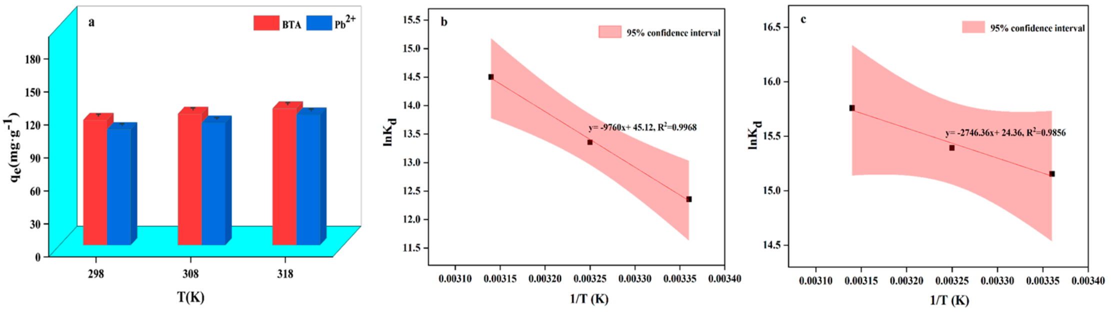

Figure S4 Effect of temperature versus WL adsorption BTA and Pb^{2+} (a); WL adsorption of BTA (b) and Pb^{2+} (c) Van't Hoff curve

Table captions

Table S1 Pore characteristics of WBC, LDH and WL
Table S2 Comparison of WL with adsorbents in other related reports
Table S3 Thermodynamic parameters of WL adsorption of BTA and Pb^{2+}

Table S1 Pore characteristics of WBC, LDH and WL

Adsorbents	Surface area ($m^2 \cdot g^{-1}$)	Pore volume ($cm^3 \cdot g^{-1}$)	Average pore size (nm)
WBC	69.251	0.116	10.612
LDH	1.933	0.097	15.575
WL	112.146	0.184	6.346

Table S2 Comparison of WL with adsorbents in other related reports

Adsorbents	T (℃)	q_m (mg·g^{-1})		References
		BTA	Pb^{2+}	
Zn-Al-O binary metal oxide	25	9.5		[1]
Zeolitic Imidazolate Framework-8	40	204.8		[2]
Zeolitic Imidazolate Framework- graphene oxide	40	207.9		[3]
Chitosan pyromellitic dianhydride modified biochar	25		9.24	[4]
Magnetic graphene oxide/MgAl-LDH	25		192.3	[5]
Sulfamic acid hydrochar	25	159.9		[6]
Hydrochar/ MgAl-LDH	25		64.4	[7]
Magnetic $MnFe_2O_4$-sludge biochar	25		174.2	[8]
WL	25	248.44	227.13	This study

Table S3 Thermodynamic parameters of WL adsorption of BTA and Pb^{2+}

Pollutants	T (K)	ΔG° (kJ mol^{-1})	ΔH° (kJ mol^{-1})	ΔS° (J mol^{-1} K^{-1})
BTA	298	-30.65	81.14	375.13
	308	-34.40		
	318	-38.15		
Pb^{2+}	298	-37.52	22.83	202.53
	308	-39.55		
	318	-41.57		

Reference

1. Xu, S.; Chen, J.; Peng, H.; Leng, S.; Li, H.; Qu, W.; Hu, Y.; Li, H.; Jiang, S.; Zhou, W.; Leng, L., Effect of biomass type and pyrolysis temperature on nitrogen in biochar, and the comparison with hydrochar. *Fuel* **2021,** 291, 120128.
2. Jiang, J.-Q.; Yang, C.-X.; Yan, X.-P., Zeolitic Imidazolate Framework-8 for Fast Adsorption and Removal of Benzotriazoles from Aqueous Solution. *ACS Applied Materials & Interfaces* **2013,** 5, (19), 9837-9842.
3. Andrew Lin, K.-Y.; Lee, W.-D., Self-assembled magnetic graphene supported ZIF-67 as a recoverable and efficient adsorbent for benzotriazole. *Chemical Engineering Journal* **2016,** 284, 1017-1027.
4. Deng, J.; Liu, Y.; Liu, S.; Zeng, G.; Tan, X.; Huang, B.; Tang, X.; Wang, S.; Hua, Q.; Yan, Z., Competitive adsorption of Pb(II), Cd(II) and Cu(II) onto chitosan-pyromellitic dianhydride modified biochar. *Journal of Colloid and Interface Science* **2017,** 506, 355-364.
5. Huang, Q.; Chen, Y.; Yu, H.; Yan, L.; Zhang, J.; Wang, B.; Du, B.; Xing, L., Magnetic graphene oxide/MgAl-layered double hydroxide nanocomposite: One-pot solvothermal synthesis, adsorption performance and mechanisms for Pb^{2+}, Cd^{2+}, and Cu^{2+}. *Chemical Engineering Journal* **2018,** 341, 1-9.
6. Deng, J.; Li, X.; Wei, X.; Liu, Y.; Liang, J.; Tang, N.; Song, B.; Chen, X.; Cheng, X., Sulfamic acid modified hydrochar derived from sawdust for removal of benzotriazole and Cu(II) from aqueous solution: Adsorption behavior and mechanism. *Bioresource Technology* **2019,** 290, 121765.
7. Luo, X.; Huang, Z.; Lin, J.; Li, X.; Qiu, J.; Liu, J.; Mao, X., Hydrothermal carbonization of sewage sludge and in-situ preparation of hydrochar/MgAl-layered double hydroxides composites for adsorption of Pb(II). *Journal of Cleaner Production* **2020,** 258, 120991.
8. Chen, Y.; Xu, F.; Li, H.; Li, Y.; Liu, Y.; Chen, Y.; Li, M.; Li, L.; Jiang, H.; Chen, L., Simple hydrothermal synthesis of magnetic MnFe2O4-sludge biochar composites for removal of aqueous Pb2+. *Journal of Analytical and Applied Pyrolysis* **2021,** 156, 105173.
